# Genome-wide identification of the ATP-dependent zinc metalloprotease (FtsH) in *Triticeae species* reveals that *TaFtsH-1* regulates cadmium tolerance in *Triticum aestivum*

**DOI:** 10.1371/journal.pone.0316486

**Published:** 2024-12-31

**Authors:** Yuxi Huang, Lifan Cao, Tanxing Chen, Xiaoqiang Chang, Yumei Fang, Liuliu Wu

**Affiliations:** 1 Henan Academy of Sciences, Zhengzhou, China; 2 College of Agriculture, Xinyang Agriculture and Forestry University, Xinyang, China; CSIR-Institute of Himalayan Bioresource Technology: Institute of Himalayan Bioresource Technology CSIR, INDIA

## Abstract

The ATP-dependent zinc metalloprotease (FtsH) protein gene family is essential for plant growth, development, and stress responses. Although *FtsH* genes have been identified in various plant species, the *FtsH* gene family in wheat (*Triticum aestivum*) remains unstudied. In this study, we identified 11 *TaFtsH* genes with uneven chromosomal distribution, significant variations in gene sequence length, and differing intron numbers among individual members. Additionally, these proteins exhibit similar physicochemical characteristics as well as secondary and tertiary structures. The *FtsH* genes can be classified into eight groups, each characterized by similar structures and conserved motifs. Intraspecific and interspecific comparisons further revealed extensive gene duplications within the *TaFtsH* gene family, indicating a closer relationship to maize. Analysis of *cis*-acting elements in the promoter regions of *TaFtsH* genes revealed developmental and stress-responsive elements in most of the genes. Expression pattern analysis showed that *TaFtsH* genes are expressed in all wheat tissues, though with varying patterns. *TaFtsH* genes displayed differential responses to CdCl_**2**_, ZnSO_**4**_, and MnSO_**4**_ stress treatments. Gene Ontology (GO) enrichment analysis indicated that *TaFtsH* genes are involved in protein hydrolysis. Barley stripe mosaic virus-induced gene silencing (BSMV-VIGS) technology confirmed the function of *TaFtsH-1*, indicating that silencing *TaFtsH-1* enhances common wheat’s resistance to cadmium (Cd) toxicity. In summary, this study offers an in-depth understanding of the *FtsH* gene family in wheat, establishing a solid basis for comprehending its functions, genetic mechanisms, and improving wheat’s tolerance to heavy metal contamination.

## 1. Introduction

Protein hydrolysis is a degradation process that provides nutrients, regulates protein levels, removes or excess proteins, and facilitates post-translational modifications and signaling [1,2]. The *FtsH* gene, encoding an ATP-dependent zinc metalloprotease, was originally discovered in *Escherichia coli*. It belongs to the AAA+ family of membrane-bound metalloproteases, which are found in eubacteria, animals, and plants [3,4]. The FtsH proteins contain three conserved structural domains and are classified within the M41 peptidase family [5,6]. They have a zinc-binding motif at the active site of the protease, which is essential for catalyzing hydrolysis [7]. Additionally, FtsH proteins participate in the assembly of hexameric complexes and provide ATPase activity to extract membrane substrates [8,9]. These complexes encapsulate the central active site for protein hydrolysis and can deactivate individual subunits [10].

Plants and cyanobacteria have a greater number of *FtsH* genes compared to non-photosynthetic organisms. Plant *FtsH* genes were first identified in spinach (*Spinacia oleracea*) leaves [11], later found in tobacco (*Nicotiana tabacum*) [12], Arabidopsis (*Arabidopsis thaliana*) [13], and rice (*Oryza sativa*) [14]. In plants, FtsH proteins act as both proteases and molecular chaperones, aiding in protein assembly and folding. Most members of this protein family are known to influence chloroplast development and homeostasis in vivo. They play roles in various biological and abiotic stresses [15]. For instance, Reducing the expression of Arabidopsis *AtFtsH2* and *AtFtsH5* led to an accumulation of reactive oxygen species, causing the bleaching of Arabidopsis leaves [16]. Similar phenotypes have been observed in other species as well. Knocking down the *NtFtsH2* and *NtFtsH1* genes in tobacco also caused the leaves to turn yellow [17]. The *OsFtsH2* knockout mutant rice exhibits albino leaves and fails to progress beyond the three-leaf stage [18]. Reduced inhibition of *NtDS9* gene expression enhanced the sensitivity of virus resistance [19]. Knocking down the *AtFtsH12* and *AtFtsHi1* genes resulted in direct embryo lethality during Arabidopsis development [20,21]. In alfalfa under low temperatures, the expression the *FtsH* gene modulates light stress [22]. In pear, *LeFstH6* is a typical heat shock gene that can interact with other heat shock gene factors [23]. *AtFtsH11* plays a crucial role in stabilizing photosystem II and cytochrome complexes at high temperatures [24,25]. In soybean, *GmFtsH9*, similar to the *AtFtsH2* gene, may be involved in regulating PSII function [26]. In pepper, silencing of the *CaFtsH1* or *CaFtsH8* genes alters normal leaf development [27]. *CaFtsH06* can be rapidly induced in response to abiotic stressors, including heat, salt, and drought [28]. Additionally, the *TaFtsH25* gene is important for improving crop yields [29].

Wheat is one of the top three primary food crops globally, providing nourishment to approximately 35% of the world’s population [30]. As a major food source, wheat absorbs both essential and non-essential elements from the soil, which raises concerns about the potential transfer of toxic elements to higher trophic levels, particularly heavy metals [31–33]. The *FtsH* gene family has been extensively investigated in various organisms, including microorganisms such as *Lactobacillus plantarum* [34], *Escherichia coli* [35], and *Bacillus subtilis* [36], as well as plants such as *Arabidopsis thaliana* [16], *Zea mays* [37], and *Oryza sativa* [18]. Taghavizadeh Yazdi et al. reported that the HSP70 family is more sensitive to Cd intoxication than other HSPs [38]. The interaction between HSP70-4 and TaFtsH-1 was detected (S1 Fig). Therefore, we established the link between the *TaFtsH* genes and Cd stress. However, there is limited research on the *FtsH* gene family in wheat, particularly concerning *FtsH* genes associated with resistance to heavy metal stress. Given the significance of the *FtsH* gene family in plant growth and development, studying these genes can help identify new resistance genes and elucidate the molecular mechanisms of stress resistance in wheat. Therefore, a comprehensive investigation of the *FtsH* gene family in wheat is crucial. This study performed a comprehensive analysis of the *TaFtsH* gene family, including gene structure, conserved motifs, chromosomal mapping, and molecular phylogenetic analysis. Sequence comparisons established the evolutionary relationships between wheat and various species, and the expression patterns of *FtsH* are examined under different heavy metal stress conditions. The role of the target gene *TaFtsH-1* in resistance to cadmium (Cd) was confirmed using barley stripe mosaic virus-induced gene silencing (BSMV-VIGS). This study is essential for enhancing our understanding of the *FtsH* gene family in wheat, especially in elucidating its role in heavy metal stress resistance.

## 2. Materials and methods

### 2.1. Materials and treatments

The wheat cultivar AK58 was utilized for expression analysis and functional characterization of *FtsH* genes.

The seeds were soaked in a 5% sodium hypochlorite (NaClO) solution for five minutes, rinsed with sterile water, and kept in the dark at 25°C for three days to germinate. The germinated seeds were then transferred to a light incubator set at 25°C with a 16-hour light/ 8-hour dark cycle and an average relative humidity of 55% for seven days. After seven days, the seedlings were provided with Hoagland nutrient solution to analyze the transcriptional levels of *TaFtsH* genes under abiotic stress conditions [39]. The seedlings were exposed to three treatments: 0.2 mM CdCl_2_, 0.2 mM ZnSO_4_, and 3 mM MnSO_4_ [40]. Samples were collected at 0, 6, 12, 24, 36, and 72 hours post-treatment. To investigate the tissue-specific expression of *TaFtsH* genes, 7-day-old seedlings were cold-treated at 4°C for 30 days. Subsequently, the seedlings were grown in agricultural soil under natural light for 4 months. Wheat tissues, including juvenile and adult roots, stems, and leaves, were sampled during both the seedling and mature stages.

Samples were immediately collected and stored at -80°C until further use. Each wheat sample was ground and then immersed in liquid nitrogen before total RNA extraction. The primer sequences used are listed in (S1 Table).

### 2.2. Identification and sequence analysis of *TaFtsH* genes in wheat

Initially, the hidden Markov model (HMM) files [41] for protease (PF06480) were obtained from the Pfam protein family database. Subsequently, Ensembl Plants acquired the complete genome data of *Triticum aestivum* [42]. The *FtsH* gene family in *Triticum aestivum* was analyzed using hmmsearch to identify genes with an E-value < 10−^5^ and similarity > 50%. The total number of *TaFtsH* gene family members was determined (S3 Table). The ProtParam program was used to predict the theoretical isoelectric point (pI), molecular weight, and amino acid composition of each TaFtsH protein [43,44]. The AlphaFold2 tool was used to predict orthologous protein models and generate their three-dimensional structures.

### 2.3. Chromosome distribution and gene structure analysis

The chromosome distribution of *TaFtsH* genes was extracted from the wheat genome annotation’s GFF3 file, and TBtools [45] was used to generate a chromosomal gene distribution map. The conserved domains of *TaFtsH* genes were analyzed using the NCBI Conserved Domain Database (CDD) software [46]. The TBtools tool was used to predict the structures of *TaFtsH* genes and determine the number of exons and introns. The program was configured with 10 motifs and default settings for other parameters. Subsequently, the motif structures were visualized using TBtools.

### 2.4. Phylogenetic analyses

The amino acid sequences of FtsH proteins from Arabidopsis and maize were retrieved from their respective genome databases (S4 Table). Phylogenetic trees were constructed using the MEGA 11.0 [47], and multiple sequence alignments were performed using CLUSTALW [48], with manual adjustments to optimize the alignment.

### 2.5. *Cis*-acting elements analyses

For each *TaFtsH* gene, the promoter sequence, located in a region 2 kb upstream of the start codon, was obtained from the annotation of GFF3 files extracted from the wheat genome. Predictions of *cis*-acting elements in the promoters were conducted using the PlantCARE database [49] (S5 Table).

### 2.6. Gene duplication and collinearity analysis

Gene duplications and collinearity relationships were analyzed using the Advanced Circos tool in TBtools. A comparative homology study of Arabidopsis and maize was conducted using the Plant Genome Duplication Database.

### 2.7. GO annotation analysis of TaFtsH proteins

Subcellular location of each *TaFtsH* member was established with the subcellular localization prediction program TargetP-2.0 (https://services.healthtech.dtu.dk/services/TargetP-2.0/) [50]. The GO annotations for TaFtsH proteins in wheat were obtained using the Gene Functional Annotation for Plants (GFAP 3.0) online tool (S6 Table) [51]. After converting p-values to −log10, the GO annotation results were visualized using the Hiplot online program.

### 2.8. RT-qPCR analysis

Total RNA was extracted from wheat roots, stems, and leaves using a TAKARA RNA extraction kit (Japan). First-strand cDNA was synthesized using the PrimeScript™ RT Reagent Kit with gDNA Eraser (TAKARA, Japan). The expression levels of *FtsH* genes in wheat were assessed via RT-qPCR using TB Green™ Premix Ex Taq™ II (TAKARA, Japan) and the ABI 7500 Real-Time PCR System. Tubulin [52] was used as the internal reference gene in this study. Transcriptional levels of *TaFtsH* genes were determined using the 2 ^−ΔΔCt^ method (S7 Table). The gene expression profile heatmap was generated using TBtools software.

### 2.9. BSMV-VIGS

The method of BSMV-VIGS was conducted according to the description by Wang et al. with some modifications [53]. To silence the target gene *TaFtsH-1*, a fragment of BSMV: *TaFtsH-1* was amplified using the corresponding primer pair (S1 Table) and inserted inversely into the γ-strain of BSMV to generate the BSMV: *TaFtsH-1* vector. An in vitro transcription kit (mMESSAGEmMACHINE T7, Invitrogen, Waltham, MA, USA) was utilized to produce the virus RNA. When the second leaves were fully extended, the leaves infected with the virus BSMV: *TaFtsH-1*. Wild type (WT) and BSMV: *γ*-injected plants were used as controls. The infected plants were placed in an environment of 22°C with a 16 h light/ 8 h dark cycle and a relative humidity of 70%. When the plants showed symptoms, 0.2 mM CdCl_2_, 0.2 mM ZnSO_4_, and 3 mM MnSO_4_ were added to the pots, respectively [54]. After 14 days, growth parameters such as leaf and root length and leaf and root dry weight were measured. Cd concentration was measured using inductively coupled plasma mass spectrometry (ICP-MS, PlasmaMS 300, Beijing, China) [55].

### 2.10. Statistical analysis

Data were collected using three biological replicates in separate studies. RT-qPCR data were analyzed using one-way analysis of variance (ANOVA) with SPSS software version 20.0 (SPSS, Inc., Chicago, IL, USA). To assess significant differences, Tukey’s test of significance [56] was performed using Microsoft Office 2010. Column charts were created using GraphPad Prism 8.0 software.

## 3. Results

### 3.1. Identification analysis of TaFtsH protein gene family members and characterization of their proteins

Understanding the function and regulatory mechanisms of genes necessitates comprehending protein families [57]. Protein sequences of Arabidopsis FtsH family members were used as bait to remove redundant sequences, conduct bioinformatics analysis, and evaluate conserved structural domains using the online tools HMMER and SMART. 11 FtsH proteins were identified and analyzed from the AK58 wheat genome (Tables 1 and S2). The proportion of gene numbers in species with varying ploidy levels correlated with ploidy levels [58]. The *FtsH* genes in wheat range in length from 2019 to 2436 bp, encoding between 673 (TaFtsH-7, TaFtsH-8, TaFtsH-9) and 812 (TaFtsH-4, TaFtsH-6) amino acids, with TaFtsH-6 having a molecular weight of 89.06 kD. The isoelectric points range from 5.6 (TaFtsH-7, TaFtsH-8) to 8.96 (TaFtsH-3). The gene family includes both acidic and basic proteins.

**Table 1:** Characterization of the *FtsH* gene family in wheat.

Gene Name	Sequence Accession	Gene Locus	Chr	Number of Amino Acid	Molecular Weight	Theoretical pI	Instability Index
***TaFtsH-1***	TraesCS3B02G393200	618678898–618683665	3B	802	87741.9	8.57	40.94
***TaFtsH-2***	TraesCS3D02G354500	465073388–465078108	3D	802	87572.85	8.9	40.72
***TaFtsH-3***	TraesCS3A02G360800	608379372–608384396	3A	802	87529.83	8.96	39.10
***TaFtsH-4***	TraesCS1B02G293000	510796906–510803198	1B	812	88986.18	7.1	34.82
***TaFtsH-5***	TraesCS1A02G283800	481648099–481655318	1A	808	88434.64	6.77	32.71
***TaFtsH-6***	TraesCS1D02G282900	381009927–381016026	1D	812	89056.27	7.1	34.15
***TaFtsH-7***	TraesCS7A02G471000	667210380–667214538	7A	673	71858.41	5.6	33.56
***TaFtsH-8***	TraesCS7D02G458400	576281486–576285471	7D	673	71871.41	5.6	33.51
***TaFtsH-9***	TraesCS7B02G373000	638881377–638885540	7B	673	71871.45	5.69	33.16
***TaFtsH-10***	TraesCS7B02G093400	106949589–106952226	7B	682	72773.41	6.22	37.51
***TaFtsH-11***	TraesCS7D02G189400	142610132–142612501	7D	682	72685.41	5.99	39.15

**Fig 1:**
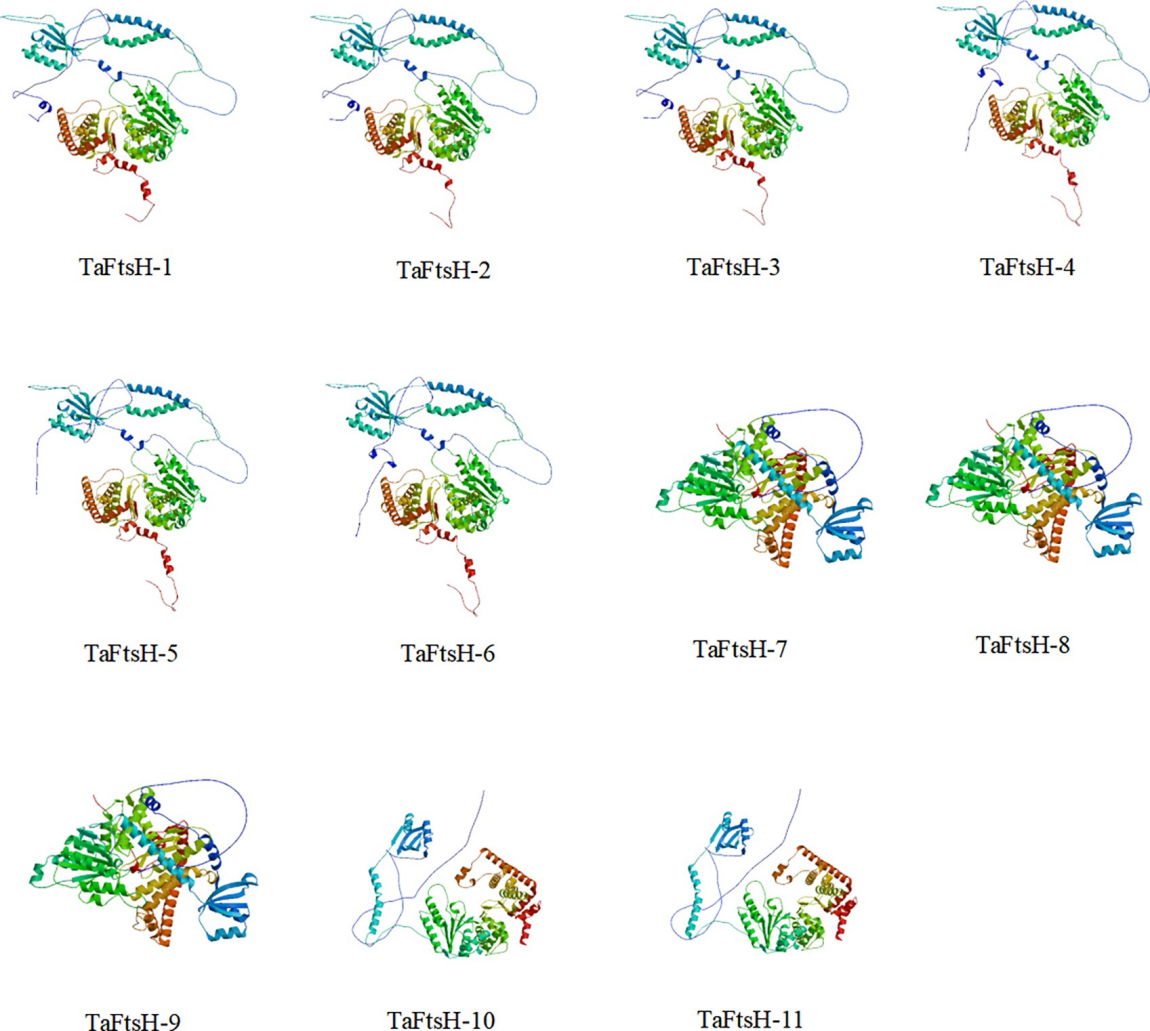
Tertiary structures of TaFtsH proteins.

The secondary structures of 11 FtsH proteins were examined, which typically consist of α-helices, extended strands, random coils, and β-turns. The findings indicated that the secondary structures of the FtsH proteins are similar, suggesting that these proteins may form comparable higher-order structures and exhibit similar activities (S8 Table). Extended strands and β-turns were the least represented, whereas α-helices (40.25%) and random coils (38.62%) comprised the largest proportions. secondary structures, such as α-helices and β-sheets, are commonly observed in peptide chains, with β-turns acting as connectors between them. A protein’s tertiary structure is formed by further twisting and folding of its secondary structure. This structure is stabilized by hydrogen bonds, hydrophobic interactions, and electrostatic interactions between the side chains of amino acids. Predicting the three-dimensional structure of a protein is crucial for understanding its mode of action. Subsequently, we used online tools to estimate the tertiary structures of the 11 FtsH protein family members (Fig 1). Our results revealed that proteins with symmetric spatial structures and identical three-dimensional conformations are primarily composed of α-helices.

### 3.2. Sequence analysis of FtsH protein family

**Fig 2:**
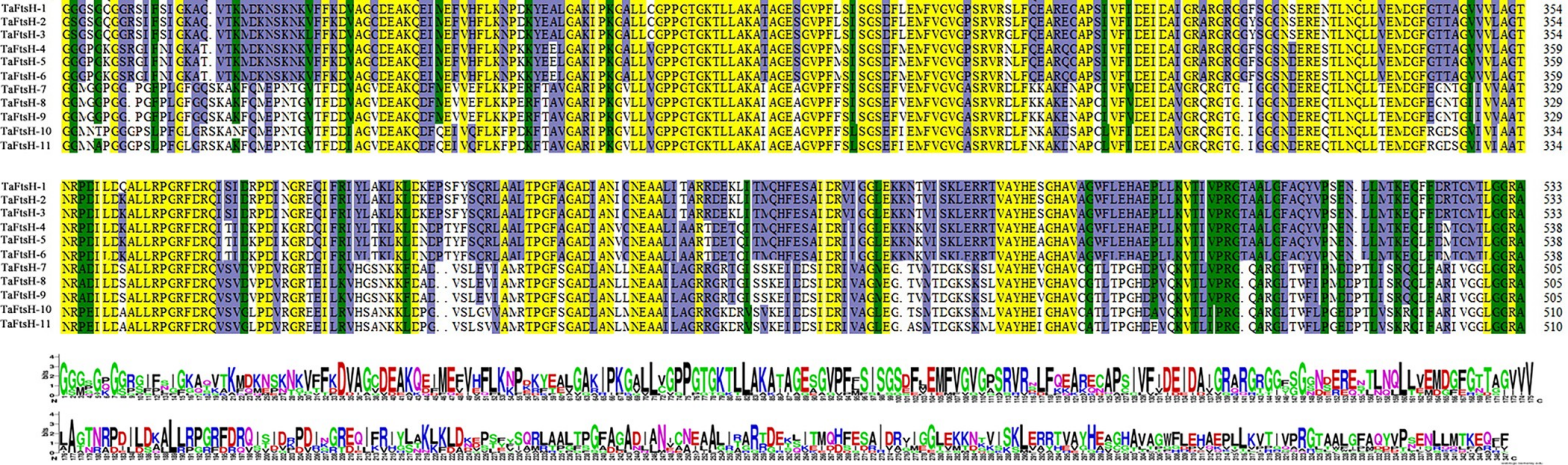
Multiple sequence comparison of the TaFtsH proteins.

11 TaFtsH proteins exhibited a high level of sequence similarity in the multiple alignment analysis conducted with DNAMAN software (Fig 2), with each protein containing a PF06480 FtsH superfamily domain. These results underscore the significant structural conservation within this protein family (Fig 2).

### 3.3. Analysis of the structures and motifs of *TaFtsH* genes

**Fig 3:**
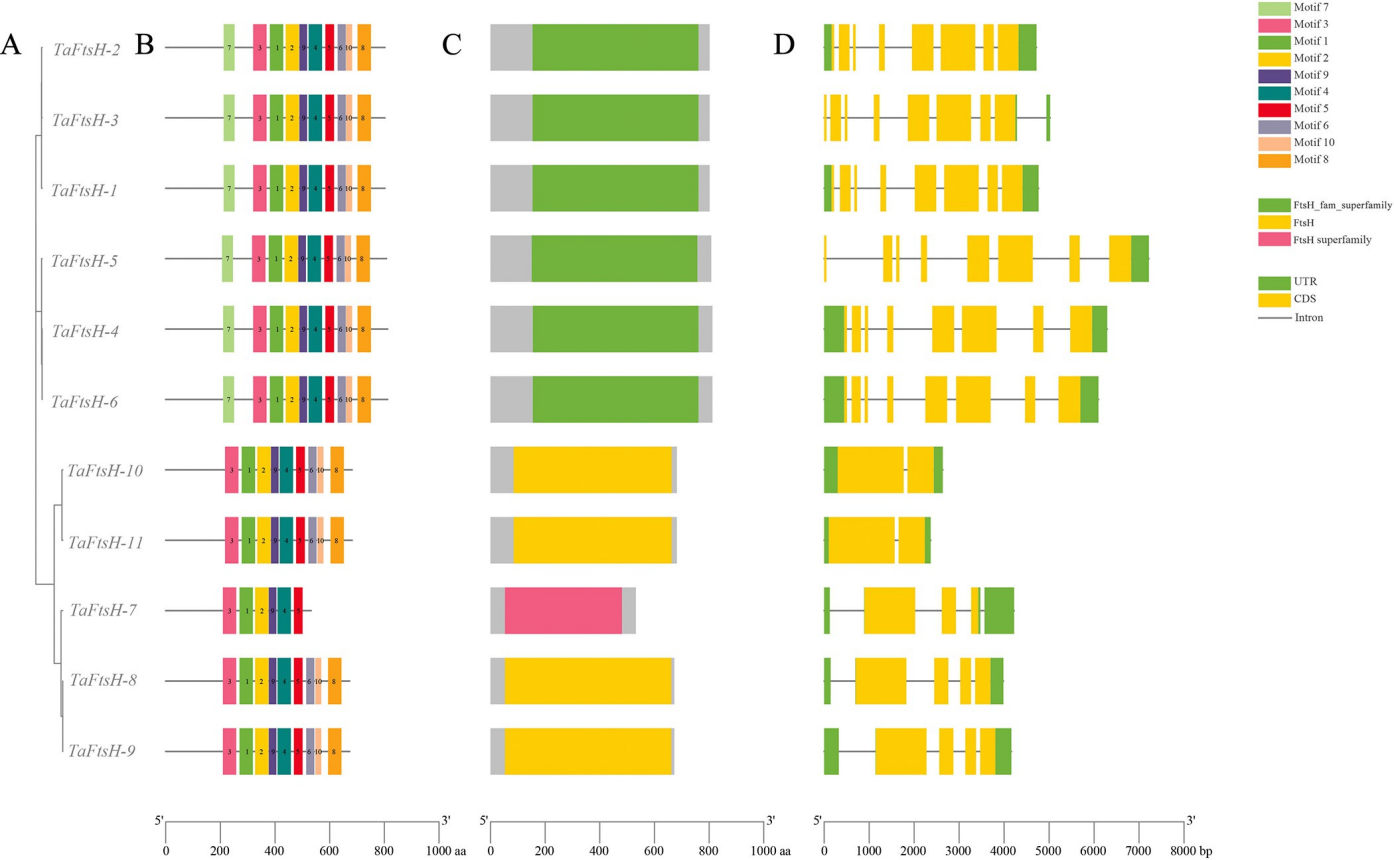
Phylogenetic relationships, conserved motifs and gene structures of 11 *TaFtsH* genes. (a) Phylogenetic tree; (b) Conserved motifs. Different colored frames represent different protein motifs; (c) Conserved domain; (d) Coding sequences (CDS) and upstream/downstream sequences are indicated by green frames, yellow frames, respectively.

The structures of the *TaFtsH* genes were comprehensively analyzed using TBtools to construct a map (Fig 3). The wheat *FtsH* family was found to comprise 10 motifs predicted by MEME software, with their types and sequences being highly similar. As homology increased, the gene motif arrangement exhibited a greater degree of similarity (Fig 3B). Each of the 11 *TaFtsH* genes contained motifs 1, 2, 3, 4, and 5. *TaFtsH-1* to *TaFtsH-6* each exhibited 10 motifs. In contrast, *TaFtsH-7* contained only motifs 1, 2, 3, 4, 5, and 9. *TaFtsH-8* to *TaFtsH-11* lacked motifs 7. Therefore, the *TaFtsH* gene sequences within the same population are highly homologous.

The *FtsH*_fam_superfamily structural domain was present in the *TaFtsH-1* to *TaFtsH-6* genes (Fig 3C). *TaFtsH-7* exclusively featured the *FtsH*_superfamily structural domain, whereas *TaFtsH-8* to *TaFtsH-11* exhibited the *FtsH* structural domain (Fig 3C).

Evolution often leads to variations in intron numbers, thereby enhancing gene diversity in both composition and functionality [59,60]. Differential exon and intron patterns are crucial for evolutionary processes. Our analysis revealed that the *FtsH* genes have 1–3 UTR regions (Fig 3D). *TaFtsH-1*, *TaFtsH-2*, *TaFtsH-3*, *TaFtsH-4*, *TaFtsH-5*, and *TaFtsH-6* each contain 8 CDSs. *TaFtsH-10* and *TaFtsH-11* each contain two CDSs. Except for *TaFtsH-5* stands out as the only one with a single UTR, the rest of the *TaFtsH* genes contain two UTRs.

### 3.4. Chromosome distribution of *FtsH* genes in wheat

**Fig 4:**
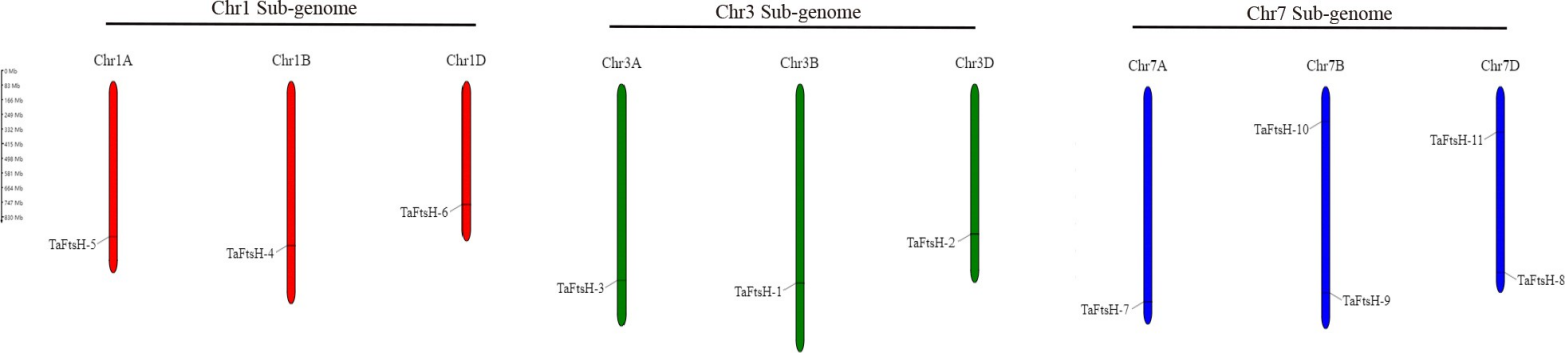
Chromosomal locations analysis of the wheat *FtsH* genes.

The genomic locations of the *FtsH* gene family members in wheat were analyzed. The study revealed that the 11 *TaFtsH* genes are unevenly distributed across nine of the 42 chromosomes (Fig 4). The most evenly distributed genes are located on chromosomes 1 and 3, with each chromosome containing three genes. Conversely, Chromosome 7 contains five genes.

### 3.5. Phylogenetic analysis of the *TaFtsH* genes

**Fig 5:**
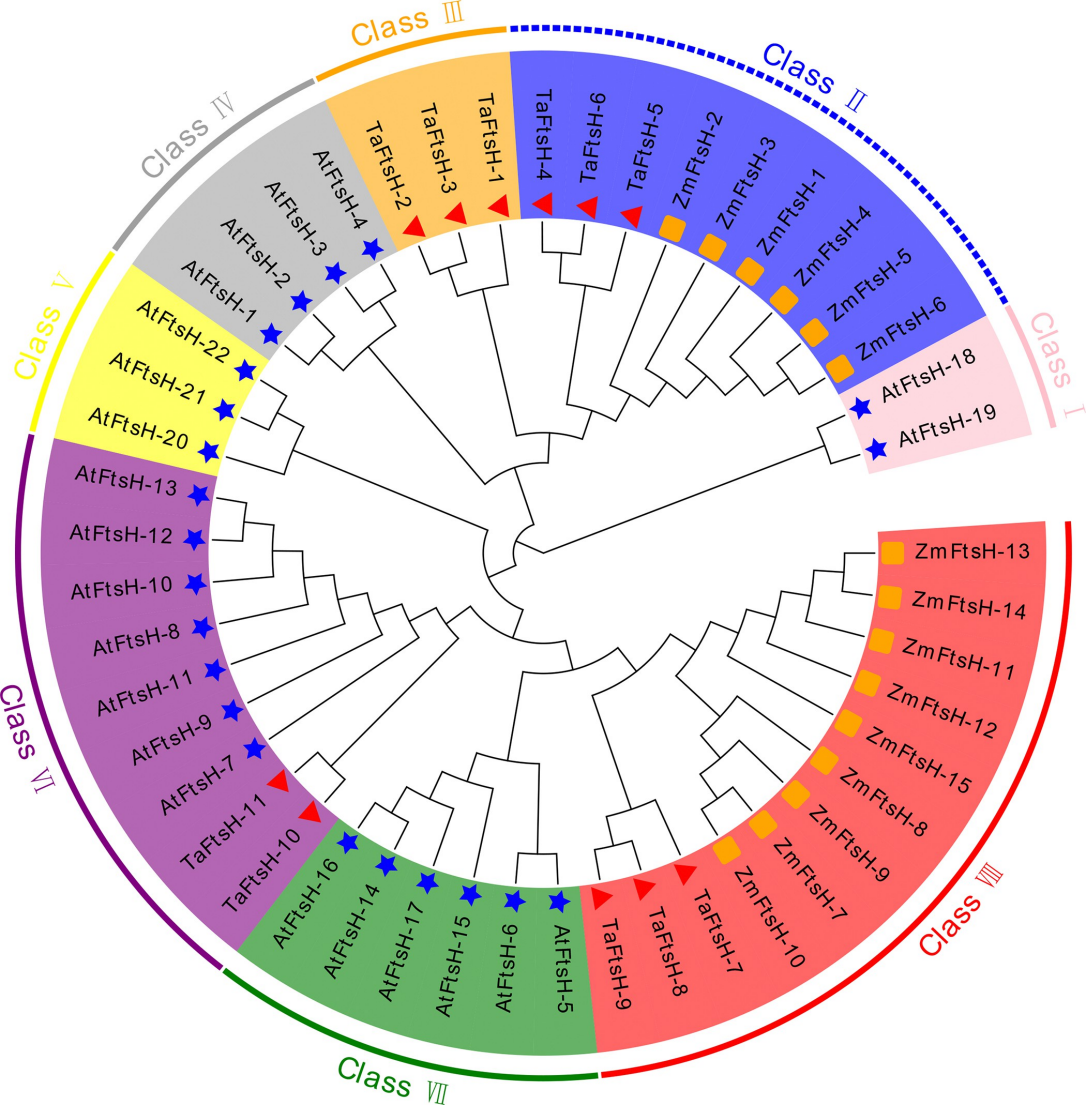
Phylogenetic analysis of *FtsH* genes in *Triticum aestivum*, *Arabidopsis thaliana* and *Zea mays*. The different shapes indicated various species. Triangle: Wheat; Star: Arabidopsis; Square: Maize. The different background colours indicated the different *FtsH* gene types.

To explore the evolutionary relationships of the *TaFtsH* genes, phylogenetic trees were constructed incorporating the *FtsH* genes from *Triticum aestivum*, *Arabidopsis thaliana* (*AtFtsH*), and *Zea mays* (*ZmFtsH*). Fig 5 shows that Arabidopsis *FtsH* genes and *TaFtsH* genes are located on separate branches with minimal interactions. The *FtsH* genes were classified into eight subfamilies based on species similarity. *TaFtsH* genes were predominantly found in classes II, III, VI, and VIII, while most *AtFtsH* genes clustered in classes I, IV, V, VI, and VII. This distribution indicates a potential similarity in evolutionary patterns for *TaFtsH* genes. *ZmFtsH* genes were assigned to classes II and VIII. Among the *TaFtsH* genes, those in classes II, III, VI, and VIII accounted for 27.3%, 27.3%, 18.2%, and 27.3% of the total number of wheat *TaFtsH* genes, respectively (Fig 5 and Table 2).

**Table 2:** Numbers of FtsH proteins in the genome of each analyzed species.

Genome	Total	Subgroup							
	Number	Class Ⅰ	Class Ⅱ	Class Ⅲ	Class Ⅳ	Class Ⅴ	Class Ⅵ	Class Ⅶ	Class Ⅷ
***Triticum aestivum***	11	0	3	3	0	0	2	0	3
***Arabidopsis thaliana***	22	2	0	0	4	3	7	6	0
***Zea mays***	15	0	6	0	0	0	0	0	9

The analysis indicated that *FtsH* genes have exhibited greater conservation throughout the evolution of monocotyledon species compared to dicotyledon species. Additionally, the *TaFtsH* genes show a closer relationship to the maize *FtsH* genes (Fig 5 and Table 2).

### 3.6. The *Cis*-acting elements analysis in the promoters of *TaFtsH* genes

**Fig 6:**
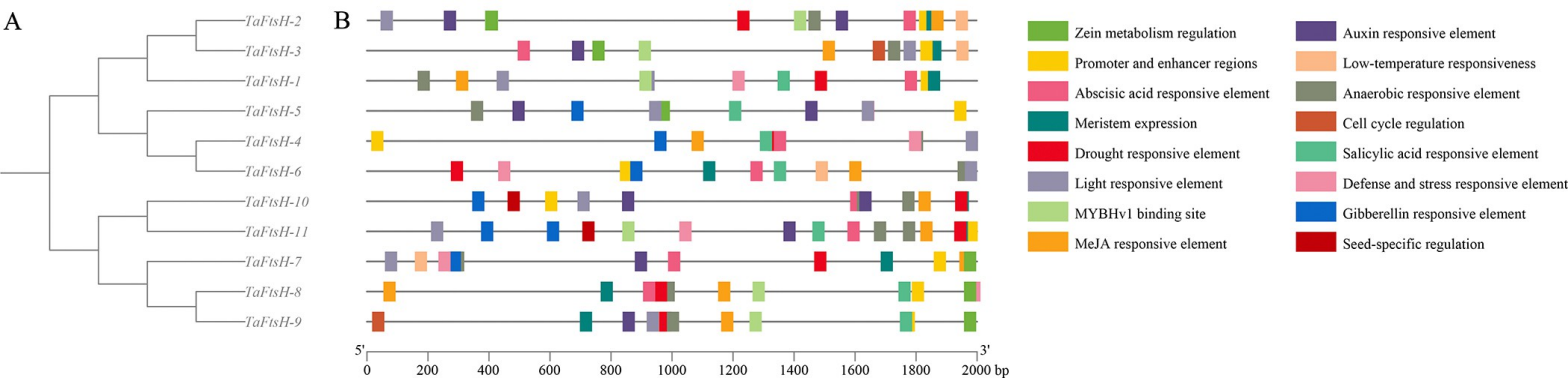
Promoter *cis*-acting elements analysis of the wheat *FtsH* genes. The different coloured boxes indicated different types of *cis*-acting elements. (a) Phylogenetic tree; (b) Promoter *cis*-acting elements.

To further investigate the regulatory mechanisms of the wheat *FtsH* gene family in response to abiotic stress, we extracted the complete 2000 bp upstream sequences of 11 *FtsH* genes from wheat genomic data and conducted *cis-*acting element analysis using the PlantCARE database (Fig 6). Sixteen elements with distinct functions were identified and characterized. These elements were categorized into four primary groups: light-responsive elements (e.g., light responsiveness), hormone-responsive elements (e.g., abscisic acid, auxin, salicylic acid MeJA, gibberellin), abiotic stress-responsive elements (e.g., defense and stress responses, drought, low temperature, anoxic-specific, anaerobic induction), and other elements associated with promoters, enhancers, zein metabolism, meristem expression, MYBHvI binding sites, cell cycle regulation, and seed specificity.

### 3.7. Collinearity analysis of *TaFtsH* genes

**Fig 7:**
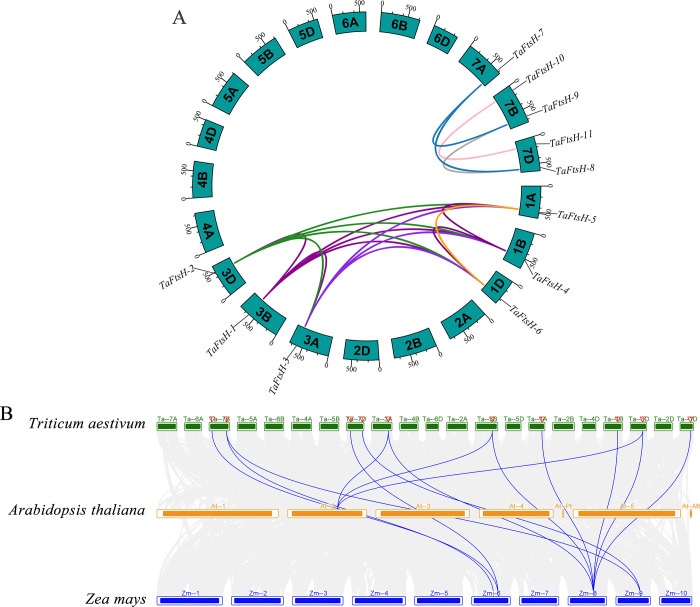
Collinearity analysis of the *FtsH* genes. (a) Collinearity analysis among *TaFtsH* genes in *Triticum aestivum*. Pairs of segmented duplicate genes are linked with different color lines; (b) Collinearity analysis of *FtsH* genes among *Triticum aestivum*, *Arabidopsis thaliana*, and *Zea mays* through comparative genome analyses. The blue lines represented the collinearity of *FtsH* gene pairs.

The collinearity of *TaFtsH* genes was analyzed to gain a better understanding of the fundamental mechanisms involved in genomic evolution. The *TaFtsH* genes are present on several homologous chromosomes and were likely formed by segmental duplication (Fig 7A). The evolutionary similarities between the two genomes are illustrated by the observation that individual chromosomes from both genomes are predominantly linked by lines of the same color (Fig 7A). Gene pairs connected by a single line are categorized as homologous. Chromosome 7 revealed the presence of segmentally duplicated gene pairs: *TaFtsH-7* and *TaFtsH-11*, *TaFtsH-7* and *TaFtsH-9*, *TaFtsH-8* and *TaFtsH-9*, and *TaFtsH-10* and *TaFtsH-11* (Fig 7A). *TaFtsH-5* and *TaFtsH-6*, *TaFtsH-5* and *TaFtsH-4*, *TaFtsH-5* and *TaFtsH-1*, *TaFtsH-5* and *TaFtsH-2*, *TaFtsH-5* and *TaFtsH-3* were shown as gene pairs.

A collinearity analysis was conducted on *Triticum aestivum*, *Arabidopsis thaliana*, and *Zea mays* to elucidate the homologous relationships of the 48 *FtsH* genes across these plant species, as shown in Fig 7B. The results indicated that the *FtsH* genes underwent selective expansion and reduction during their evolution. 14 *FtsH* genes were homologous with Arabidopsis, while only thirteen were homologous with maize. 11 *FtsH* genes showed homology with both Arabidopsis and maize (Fig 7B), suggesting a high level of evolutionary conservation. These genes likely played a pivotal role in the evolution of the *FtsH* gene family. To determine the genetic links between species and predict gene functions, a comparative study of *FtsH* genes between wheat and other plants is essential.

### 3.8. The Gene Ontology (GO) annotation analysis of TaFtsH proteins

**Fig 8:**
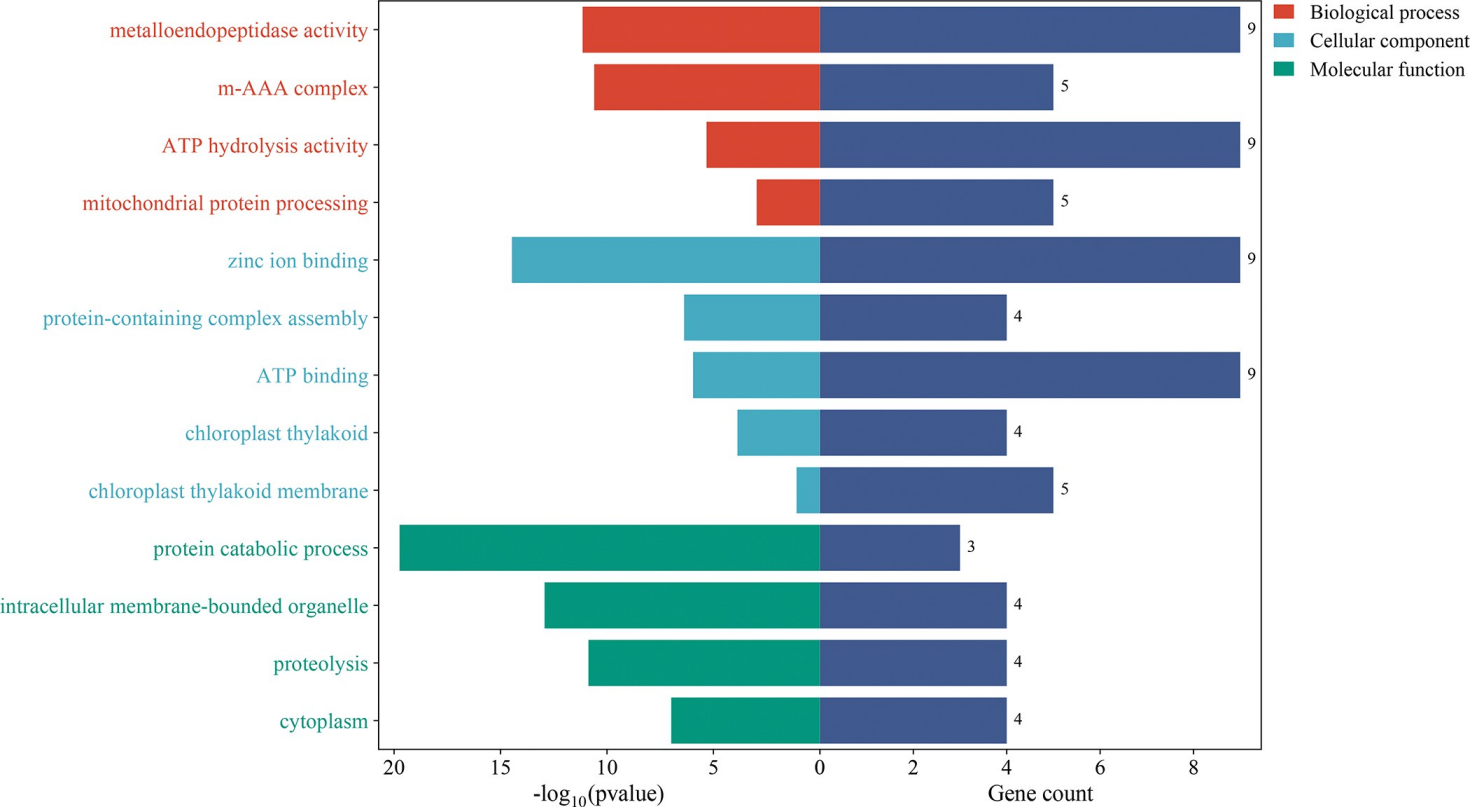
GO annotation analysis of the *Triticum aestivum FtsH* genes. Green column represented the molecular function; Wathet blue column represented the cellular component; Orange column represented the biological process and dark blue column represented the number of *TaFtsH* genes.

GO annotation studies revealed that the 11 TaFtsH proteins participate in diverse biological processes, cellular components, and molecular functions (Fig 8). These findings suggest that TaFtsH proteins are involved in a variety of molecular and biological processes (Fig 6 and S6 Table). Analysis of the biological processes mediated by these proteins revealed that TaFtsH proteins play a central role in mitochondrial protein processing, proteolysis, and the assembly of macromolecular complexes. Furthermore, all proteins, with the exception of *TaFtsH-6*, are located within the chloroplast. Examination of cellular components showed that TaFtsH proteins are predominantly located in intracellular organelles such as chloroplasts, plastids, and integral membrane components. TaFtsH proteins are also involved in various molecular functions, including ATP-dependent peptidase and metalloendopeptidase activities, ATP and zinc ion binding, and ATPase activity.

### 3.9. Expression levels of *TaFtsH* genes in tissues

**Fig 9:**
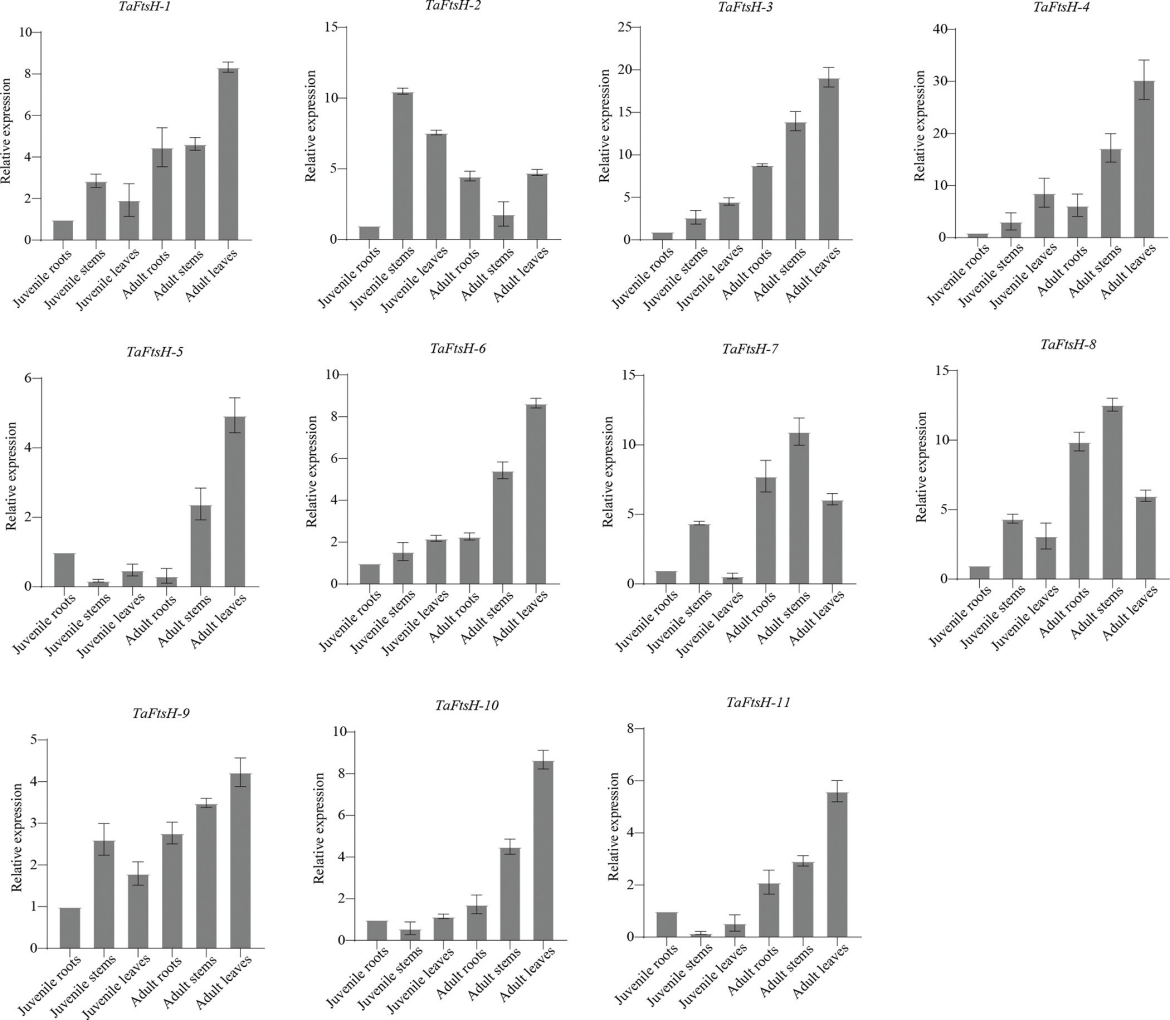
Expression patterns analysis of *TaFtsH* genes in different tissues.

The expression levels of 11 *TaFtsH* genes were analyzed across various wheat tissues and organs (Fig 9). The expression of *TaFtsH* gene family members varied among different tissues. *TaFtsH-1*, *TaFtsH-3*, *TaFtsH-4*, *TaFtsH-6* and *TaFtsH-9* exhibited the highest expression levels in adult leaves and the lowest in juvenile roots. *TaFtsH-2* showed higher expression levels in juvenile wheat stems, whereas *TaFtsH-5*, *TaFtsH-10*, and *TaFtsH-11* showed the lowest expression levels. *TaFtsH-8* exhibited the highest levels in adult stems.

### 3.10. Expression patterns of *TaFtsH* genes under various metal stresses

**Fig 10:**
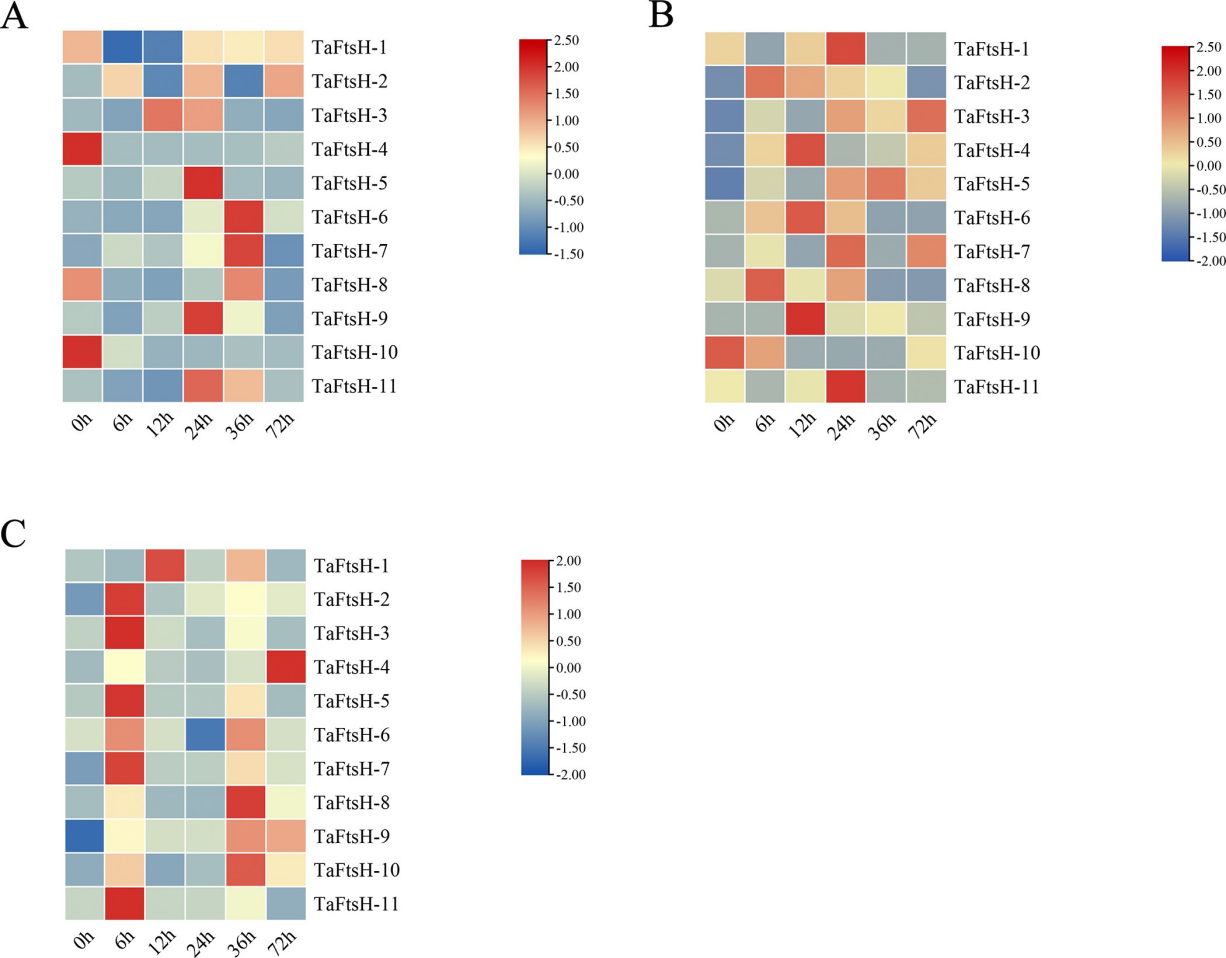
Expression levels analysis of *TaFtsH* genes in response to different stresses. (a) 0.2 mM CdCl_2_ treatment; (b) 0.2 mM ZnSO_4_ treatment; (c) 3 mM MnSO_4_ treatment. The colour gradient (orange/yellow/blue) represented the gene expression level (from high to low).

The expression patterns of *TaFtsH* genes under metal stress conditions (0.2 mM CdCl_2_, 0.2 mM ZnSO_4_, and 3 mM MnSO_4_) were investigated by performing RT-qPCR (Real-Time Quantitative PCR). Under CdCl_2_ stress, the expression levels of *TaFtsH-1*, *TaFtsH-2*, *TaFtsH-5*, *TaFtsH-9*, and *TaFtsH-11* peaked at 24 hours (Fig 10A). While the expression levels of *TaFtsH-5* and *TaFtsH-8* dramatically increased and then decreased at 72 hours; the expression levels of *TaFtsH-1*, *TaFtsH-4*, *TaFtsH-10*, and *TaFtsH-11* first increased and then decreased, and then increased. The expression levels of 5 out of the 11 *TaFtsH* genes (*TaFtsH-2*, *TaFtsH-3*, *TaFtsH-4*, *TaFtsH-5*, and *TaFtsH-9*) were generally upregulated over time under ZnSO_4_ stress. Conversely, *TaFtsH-2*, *TaFtsH-7*, *TaFtsH-9*, *TaFtsH-10*, and *TaFtsH-11* significantly increased and then decreased after 12 hours of MnSO_4_ treatment. At 72 hours, *TaFtsH-4* exhibited the highest expression levels (Fig 10C).

### 3.11. *TaFtsH-1* negatively regulate Cd resistance in wheat

**Fig 11:**
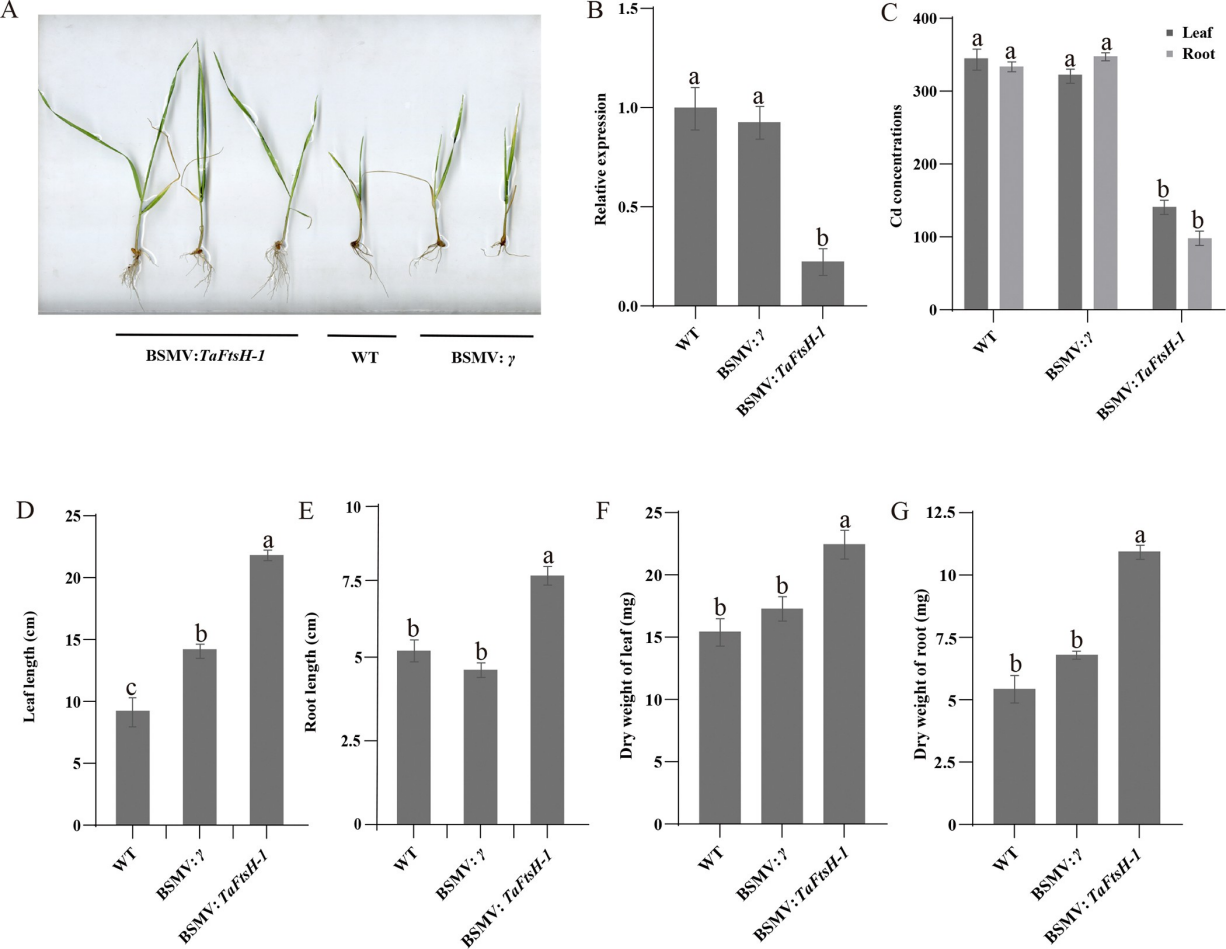
Function analysis of the *TaFtsH-1* gene by BSMV-VIGS. (a) Phenotypes of WT, BSMV: *γ*-injected plants and BSMV: *TaFtsH-1*-injected plants; (b) Rrelative expression; (c) Cd concentrations; (d) Leaf length; (e) Root length; (f) Leaf dry weight; (g) Root dry weight. Varied letters (a-e) indicated a significant difference (p < 0.05).

*TaFtsH-1* expression levels were reduced by 74% and 76% in BSMV: *TaFtsH-1*-injected plants compared to the control (Fig 11B). The leaf/ root growth length and dry weight of BSMV: *TaFtsH-1*-injected plants treated with 0.2 mM CdCl_2_ were significantly different from those of the control (Fig 11D–11G). The length of the leaves and roots grew by 38.5%-42.3% and 15.8%-17.6%, respectively; The relative dry weight of the roots and leaves of BSMV: *TaFtsH-1*-injected plants increased by 45.9%-52.1% and 12.1%-14.7%, respectively, in comparison to WT. However, no significant differences were observed between the leaves and roots of BSMV: *TaFtsH-1*-injected plants under ZnSO_4_ and MnSO_4_ stress (S2C–S2F and S3C–S3F Figs). Cd concentration in the leaves and roots of BSMV: *TaFtsH-1*-injected plants was reduced by 60.7%, 58.8% and 69.3%, 71.1% respectively, compared to the control (Fig 11C); In contrast, the concentrations of Zn and Mg showed no significant changes (S2B and S3B Figs).

## 4. Discussion

The *FtsH* genes are ubiquitous in plant species and play a crucial role in their growth and development, particularly in chloroplasts and mitochondria [61,62]. Extensive research has been conducted on these genes in various plant species, including tobacco [12], tomato [63], potato [64], Arabidopsis [13], and maize [37]. Genome-wide analyses have revealed extensive studies on *FtsH* genes across a wide range of species. This study identified 11 *FtsH* genes in the wheat genome, named based on their chromosomal distribution, ranging from *TaFtsH-1* to *TaFtsH-11*. Despite variations in genome size among, different numbers of *FtsH* genes have been identified in various plants. For instance, 20 *FtsH* genes have been identified in tobacco [12], 12 *FtsH* in Arabidopsis [65], and 34 *FtsH* in soybean [66]. This suggests that the number of *FtsH* genes is relatively stable and not directly correlated with genome size.

Gene families containing intron-deficient genes are more likely to be involved in epigenetic processes and contribute to plant development throughout plant evolution. Exploring the origins, evolutionary paths, and potential roles of intronless and intron-deficient subfamilies can enhance our understanding of plant genome evolution and gene functional diversification [67]. Characterizing the structure of wheat genes revealed that introns are widely present in the *FtsH* genes. The *TaFtsH* genes exhibited one to eight introns. *TaFtsH* genes grouped together on the phylogenetic tree exhibited similar intron-exon structures and conserved domains, indicating their similar roles in plant function. Particularly, *TaFtsH* gene members exhibited structural and conserved domain similarities with Arabidopsis and maize, suggesting a high degree of conservation among species. Metalloproteases are a group of enzymes that play crucial roles in various biological processes. A metalloprotease gene family identified in sugarcane comprises matrix metalloproteases, zincins, inverzincins, and ATP-dependent metalloproteases [68]. These findings highlight the presence of ATP-dependent metalloproteases in plants, indicating their importance in plant biology. Subsequent research by Sun et al. investigated a novel, conserved membrane FtsH family, which comprises genes from Solanaceae and other plant species [69]. The study identified a unique group of FtsH proteins that are conserved across multiple plant species and cyanobacteria. Yoshioka and Yamamoto proposed a mechanism for the activation of FtsH proteases, emphasizing their role in the degradation of the D1 protein under light stress [61]. Collectively, these findings emphasize the evolutionary significance and functional importance of FtsH proteins in plants. The identification and characterization of FtsH proteins across different plants emphasize their critical roles in plant physiology and stress responses. Additionally, many *TaFtsH* genes contain *FtsH*-related conserved domains. A systematic phylogenetic analysis was conducted on the *FtsH* gene family in wheat. These members were classified into eight clusters, corresponding to the grouping of *FtsH* genes in soybean, pears, and Arabidopsis [23,65,66]. Gene structure and conserved motif analyses further confirmed the high homology of *TaFtsH* gene sequences within the same population. Furthermore, certain *TaFtsH* genes contained the *FtsH*_ext structural domain at the N-terminus, potentially indicating a modification in the function of the *TaFtsH* genes. The maintenance of the fundamental functions of gene families depends on the retention of *FtsH* structural domains, as diversity can alleviate selection pressures during evolution [70].

Gene duplication is a key process influencing biological evolution and a critical factor in the expansion of gene families. The *TaFtsH* genes were located on only 9 out of the 42 chromosomes, and no clear connection was found between their distribution and gene density on the chromosomes. This suggests that gene deletions during evolution have impacted the *TaFtsH* gene family. It is possible that duplication events gave rise to some of the *FtsH* genes, suggesting that the rapid development of the *FtsH* family in plants was significantly influenced by these events.

The Gene Ontology (GO) framework offers valuable insights into gene function. Analyzing differential genes using GO analysis can reveal enriched GO categories [71]. GO enrichment analysis was conducted on TaFtsH proteins to identify potential alterations in gene function associated with differential genes across various samples. According to GO annotation results, the majority of TaFtsH proteins are in chloroplasts, mitochondria, and as constituents of significant organelles such as cell membranes. Within plant organisms, these proteins participate in various biological processes, including energy metabolism, protein hydrolysis, digestion, and proteolytic metabolism, all of which are interconnected with photosynthesis.

The TaFtsH proteins likely perform enzymatic protein hydrolysis within chloroplast-like vesicles, similar to the molecular functions of FtsH proteins in Arabidopsis [72] and rice [14]. These studies indicate that TaFtsH proteins might be involved in protein hydrolysis processes in chloroplasts, potentially affecting photosynthesis in leaves and, consequently, influencing the plant growth environment. Further research involving molecular functional analysis, cell composition analysis, and biological process analysis has demonstrated that TaFtsH proteins exhibit ATP-dependent serine protease activity, metallopeptidase activity, and chlorophyll protein transport enzyme activity. This also confirmed that TaFtsH proteins are structurally similar to those in other plants, such as soybean [66], Arabidopsis [65], blue-green algae [34], and tobacco [12], which may result in similarities in their molecular functions. For example, downregulating the expression of *NtFtsH1* and *NtFtsH2* reduced the degradation of PSII-associated light-harvesting complexes, resulting in vesicle membrane damage and heterochromatin development in tobacco leaves [17]. In sulfur- or nitrogen-deficient cyanobacteria, the FtsH protein degrades the photosynthetic complex and protein quality under nutrient deprivation [15]. Additionally, the AtFtsH proteins are involved in Arabidopsis embryonic development, causing growth lethality in Arabidopsis seedlings [21,73].

Analyzing gene expression profiles enhances our understanding of the potential biological functions of genes. To elucidate the functions of *TaFtsH* genes, we examined their expression profiles across various tissues. All 11 *TaFtsH* genes were detected in every organ analyzed, with adult roots, stems, and leaves exhibiting significant expression levels, suggesting their involvement in plant growth and development. These findings are consistent with those observed in maize and tobacco [12,37]. The expression patterns of the 11 *TaFtsH* genes under three different heavy metal stress conditions were also analyzed. The results suggest that the expression levels of most *TaFtsH* genes vary under different heavy metal stress conditions. The expression of *TaFtsH-6* and *TaFtsH-9* initially increased and then decreased under both CdCl_2_ and MnSO_4_ stress; however, their expression gradually increased under ZnSO_4_ stress. Under CdCl_2_ and ZnSO_4_ stress, the expression of *TaFtsH-11* initially decreased, then increased, and finally decreased gradually. The experimental results were consistent with the response of *TaFtsH-6* under ZnSO_4_ and MnSO_4_ stress. These findings demonstrate the involvement of *FtsH* genes in the plant response to heavy metal stress. Previous studies have shown that cadmium and zinc upregulate the VAR1 gene, which encodes *FtsH5* [74]. *Aegilops tauschii FtsH-like* is associated with cadmium tolerance [75]. The *TaFtsH* genes are also important regulatory proteins in plant response to stress. Both drought and heat stress increase the expression level of the *TaFtsH6* gene in root and leaf tissues [76]. The *FtsH2/8* gene responds to salt stress in wheat [77]. The *FtsH2* and *FtsH5* genes are also identified as responding to heat stress in wheat [78]. These results are consistent with *cis-*acting element analysis, indicating that *TaFtsH* genes contain numerous *cis-*acting elements associated with a wide range of stresses. Under stress conditions, these *cis-*acting elements and their associated transcription factors can be triggered to modulate the expression of *TaFtsH* genes. Therefore, the *TaFtsH* genes may function similarly to other plant *FtsH* genes. Further research can verify the function of the *TaFtsH* genes, providing a vital foundation for deeper investigation into the genetic mechanisms and evolutionary history of the *FtsH* gene family. This study found that *TaFtsH-1* expression changes in response to Cd stress (Fig 10). Functional validation analysis demonstrated that silencing the *TaFtsH-1* gene improves resistance to Cd without significantly affecting resistance to Mg and Zn (Fig 11, S2 and S3 Figs). Under Cd stress, compared to the silenced lines, the WT displayed significantly greater growth inhibition in both plant length and dry weight. These findings suggest that *TaFtsH-1* plays a significant role as a potential negative regulator of plant growth. Additionally, Tang et al. found that genes involved in photosynthesis, electron transport, and ATP synthesis, including ATP-dependent metalloproteinases, were up-regulated in response to Zn-Cd stress in the Zn-Cd accumulating plant *Sedum alfredii* [74]. The activity of the FtsH proteins are likely crucial for the development and maintenance of chloroplast function, as well as for the repair and renewal of photosynthetic proteins impacted by Cd stress [79]. Therefore, we hypothesize that the analyzed *TaFtsH* genes might be related to Cd resistance in wheat. The other functions of these genes need to be confirmed by further analysis.

## 5. Conclusion

This study performed a detailed analysis of the wheat *FtsH* gene family. The 48 *FtsH* genes were classified into eight groups based on classification and evolutionary relationships. *TaFtsH* genes within the same evolutionary branch demonstrated significant similarity in exon and intron structure, composition, physicochemical characteristics, and secondary and tertiary structures. Comparing the homology and phylogenetic relationships of *FtsH* genes from diverse plant species provided essential information about the evolutionary characteristics of *FtsH* genes. The expression profiles of *TaFtsH* genes across various tissues and under different heavy metal treatments highlight their significant roles in plant growth, development, and abiotic stress resistance. Functional analysis indicated that silencing the *TaFtsH-1* gene enhances resistance to cadmium (Cd) toxicity in common wheat. The findings of this study offer valuable insights that enhance the comprehension of the biological functions of *TaFtsH* genes in wheat.

## Supporting information

S1 Table Primer sets used in this study. (XLS)

S2 Table Names and references for each member of the *TaFstH* gene family. (XLS)

S3 Table The sequence of TaFtsH proteins. (XLS)

S4 Table The sequence of AtFtsH and ZmFtsH proteins. (XLS)

S5 Table Predictions of cis-acting elements in the promoters. (XLS)

S6 Table The data of GO enrichment. (XLS)

S7 Table Raw qPCR data. (XLS)

S8 Table Wheat FtsH protein secondary structure main component ratio. (XLS)

S1 Fig Protein-protein interaction network for TaFstHs based on their orthologs in wheat. (JPG)

S2 Fig Function analysis of the *TaFtsH-1* gene by BSMV-VIGS under Mg stress. (a) Phenotypes of WT, BSMV: γ-injected plants and BSMV: *TaFtsH-1*-injected plants; (b) Mg concentrations; (c) Leaf length; (d) Root length; (e) Leaf dry weight; (f) Root dry weight.
(JPG)

S3 Fig Function analysis of the *TaFtsH-1* gene by BSMV-VIGS under Zn stress. (a) Phenotypes of WT, BSMV: γ-injected plants and BSMV: *TaFtsH-1*-injected plants; (b) Zn concentrations; (c) Leaf length; (d) Root length; (e) Leaf dry weight; (f) Root dry weight.
(JPG)
