# Supplementary figures and images for: Genome-wide identification of the ATP-dependent zinc metalloprotease (FtsH) in Triticeae species reveals that TaFtsH-1 regulates cadmium tolerance in Triticum aestivum
Source: PLoS One. 2024 Dec 31;19(12):e0316486. doi: 10.1371/journal.pone.0316486 (PMC11687710; doi:10.1371/journal.pone.0316486)

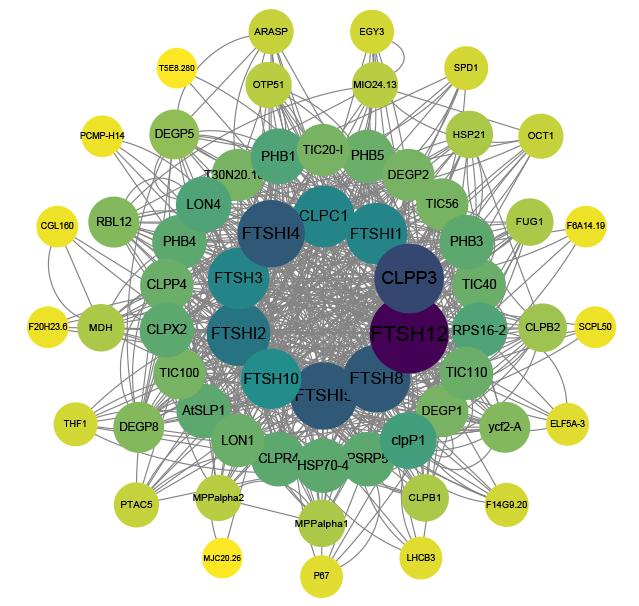

Supplement: S1 Fig — (JPG) [file pone.0316486.s009.jpg]

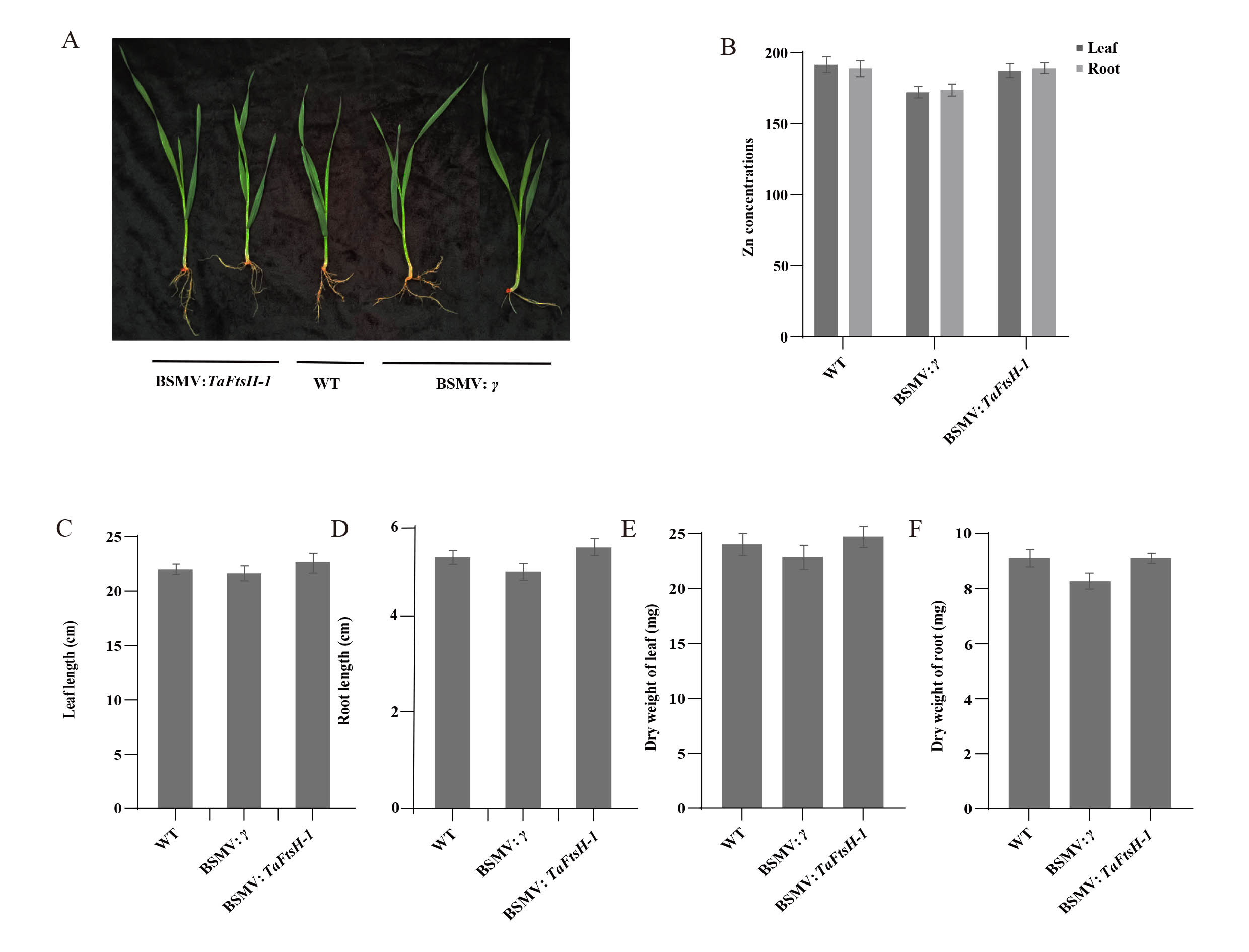

Supplement: S2 Fig — (a) Phenotypes of WT, BSMV: γ-injected plants and BSMV: TaFtsH-1-injected plants; (b) Mg concentrations; (c) Leaf length; (d) Root length; (e) Leaf dry weight; (f) Root dry weight. (JPG) [file pone.0316486.s010.jpg]

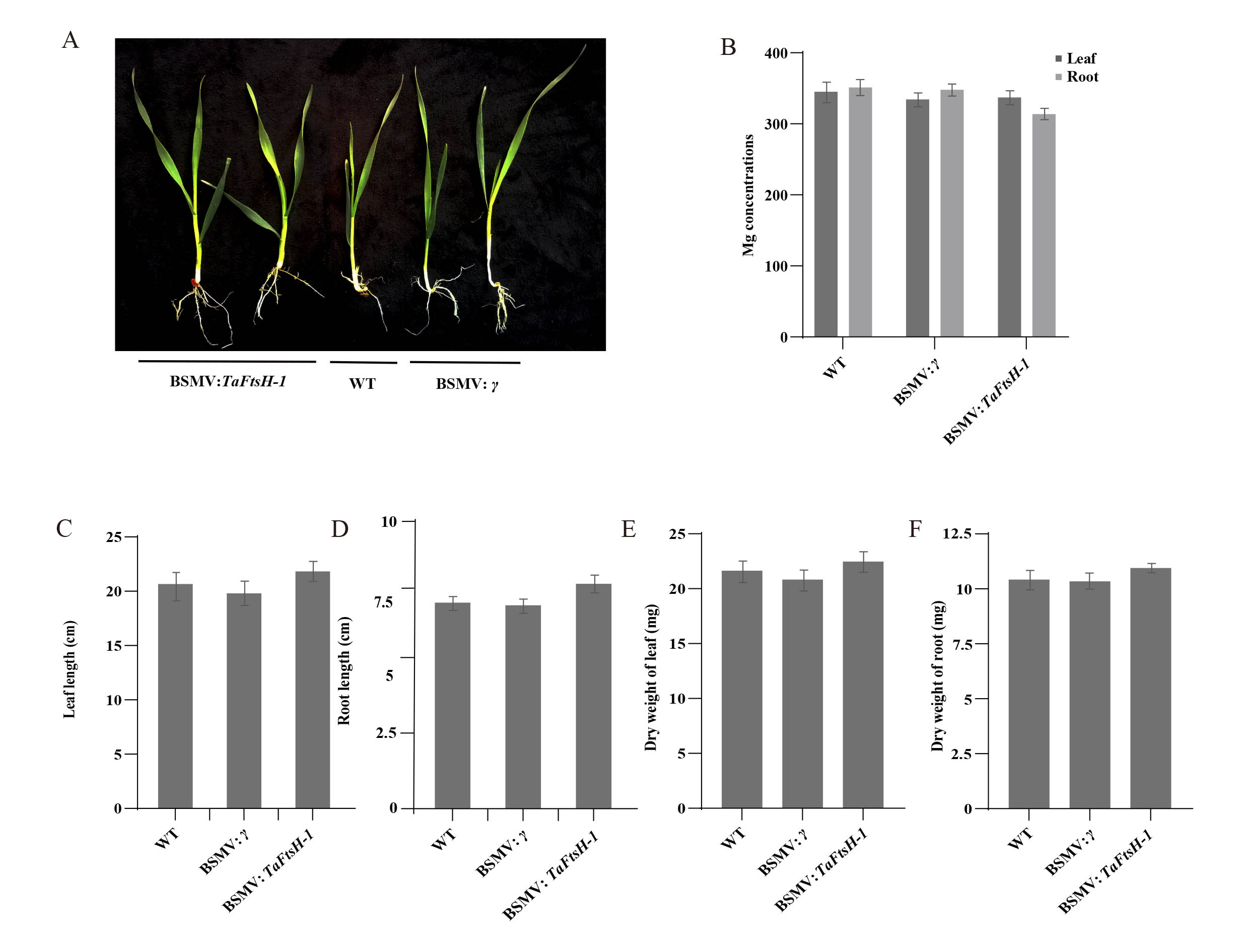

Supplement: S3 Fig — (a) Phenotypes of WT, BSMV: γ-injected plants and BSMV: TaFtsH-1-injected plants; (b) Zn concentrations; (c) Leaf length; (d) Root length; (e) Leaf dry weight; (f) Root dry weight. (JPG) [file pone.0316486.s011.jpg]
